# Automated finite element approach to generate anatomical patient-specific biomechanical models of atherosclerotic arteries from virtual histology-intravascular ultrasound

**DOI:** 10.3389/fmedt.2022.1008540

**Published:** 2022-11-29

**Authors:** Jeremy L. Warren, John E. Yoo, Clark A. Meyer, David S. Molony, Habib Samady, Heather N. Hayenga

**Affiliations:** ^1^ Department of Bioengineering, University of Texas at Dallas, Richardson, TX, United States; ^2^ Northeast Georgia Health System, Georgia Heart Institute, Gainesville, GA, United States

**Keywords:** coronary artery disease (CAD), computational modeling, cardiovascular, finite element analysis (FEA), FEBio, biomechanics

## Abstract

Despite advancements in early detection and treatment, atherosclerosis remains the leading cause of death across all cardiovascular diseases (CVD). Biomechanical analysis of atherosclerotic lesions has the potential to reveal biomechanically instable or rupture-prone regions. Treatment decisions rarely consider the biomechanics of the stenosed lesion due in-part to difficulties in obtaining this information in a clinical setting. Previous 3D FEA approaches have incompletely incorporated the complex curvature of arterial geometry, material heterogeneity, and use of patient-specific data. To address these limitations and clinical need, herein we present a user-friendly fully automated program to reconstruct and simulate the wall mechanics of patient-specific atherosclerotic coronary arteries. The program enables 3D reconstruction from patient-specific data with heterogenous tissue assignment and complex arterial curvature. Eleven arteries with coronary artery disease (CAD) underwent baseline and 6-month follow-up angiographic and virtual histology-intravascular ultrasound (VH-IVUS) imaging. VH-IVUS images were processed to remove background noise, extract VH plaque material data, and luminal and outer contours. Angiography data was used to orient the artery profiles along the 3D centerlines. The resulting surface mesh is then resampled for uniformity and tetrahedralized to generate the volumetric mesh using TetGen. A mesh convergence study revealed edge lengths between 0.04 mm and 0.2 mm produced constituent volumes that were largely unchanged, hence, to save computational resources, a value of 0.2 mm was used throughout. Materials are assigned and finite element analysis (FEA) is then performed to determine stresses and strains across the artery wall. In a representative artery, the highest average effective stress was in calcium elements with 235 kPa while necrotic elements had the lowest average stress, reaching as low as 0.79 kPa. After applying nodal smoothening, the maximum effective stress across 11 arteries remained below 288 kPa, implying biomechanically stable plaques. Indeed, all atherosclerotic plaques remained unruptured at the 6-month longitudinal follow up diagnosis. These results suggest our automated analysis may facilitate assessment of atherosclerotic plaque stability.

## Introduction

Understanding the adverse sequelae of atherosclerotic plaques is critical, as plaques are at the root of many cardiovascular illnesses (1). Plaque size, composition, blood flow, and calcium accumulation can all be detected using current imaging techniques. Current methods for assessing the risk of a major adverse cardiac event (MACE) include the Framingham Risk Score (FRS), Coronary Artery Calcium (CAC) score, Carotid Intima-Medial Thickness (CIMT), and Fractional Flow Reserve (FFR). However, these methods do not account for the structural mechanics of the artery wall, which influences acute instability risk as well as growth and remodeling of atherosclerotic plaque. Our novel method aims to address this deficit by modelling the biomechanics of a patient-specific artery.

In the last few decades, characteristics contributing to the instability of plaques have been established (2–4). Structurally, rupture-prone characteristics include thin-fibrous caps (<65 µm in thickness), large lipid core, and calcified nodules near the lumen. Characteristics defining erosion-prone areas include intimal thickening and fibrous atheroma with little/no lipid core (3, 5). Spatial correlation between plaque instability and areas of high mechanical stress that exceeds the strength of the artery were previously found (6). Maximum stress values >300 kPa in the plaque cap have been linked to follow-up rupture locations in several investigations (6–8). Ultimately, quantifying the structural mechanics of patient-specific plaques and stresses experienced by plaque constituents is a better indicator of rupture potential than size alone.

Image-based computational approaches allow for personalized risk assessment and enhanced treatment planning. Herein we present an automated method to go from 3D reconstruction of coronary artery meshes to execution and FE mechanical analysis. High-resolution imaging data can now be used to construct and evaluate comprehensive 3-D patient-specific models of arterial vasculature. Previously, research in this area was done using 2-D cross-sectional VH-IVUS images of arterial tissue or linear modeling (9, 10). Advances in medical imaging and computational approaches have paved the way for researchers to further investigate structural loading occurring in a 3-D patient-specific model. Recent approaches modelling arterial biomechanics using volumetric geometry have been successful in utilizing various imaging modalities and material models yet were limited in scope. That is they either use a limited set of materials or a single homogenized material to represent plaque constituents (11, 12), require additional manual segmentation (13, 14), and neglect arterial curvature, branching or bifurcations (12–14). In short, previous approaches do not fully address the inherent heterogeneous nature of atherosclerotic artery tissue or the complex curvature presented by coronary artery geometry (11). The publicly available automated method presented herein embraces heterogenous tissue variability and leverages patient-specific data to build an accurate 3D representation of the patient's atherosclerotic artery. The outcomes from this model seek to provide a diagnostic risk-assessment tool to assess the mechanical integrity of a patient-specific atherosclerotic artery.

## Materials and methods

**Figure 1:**
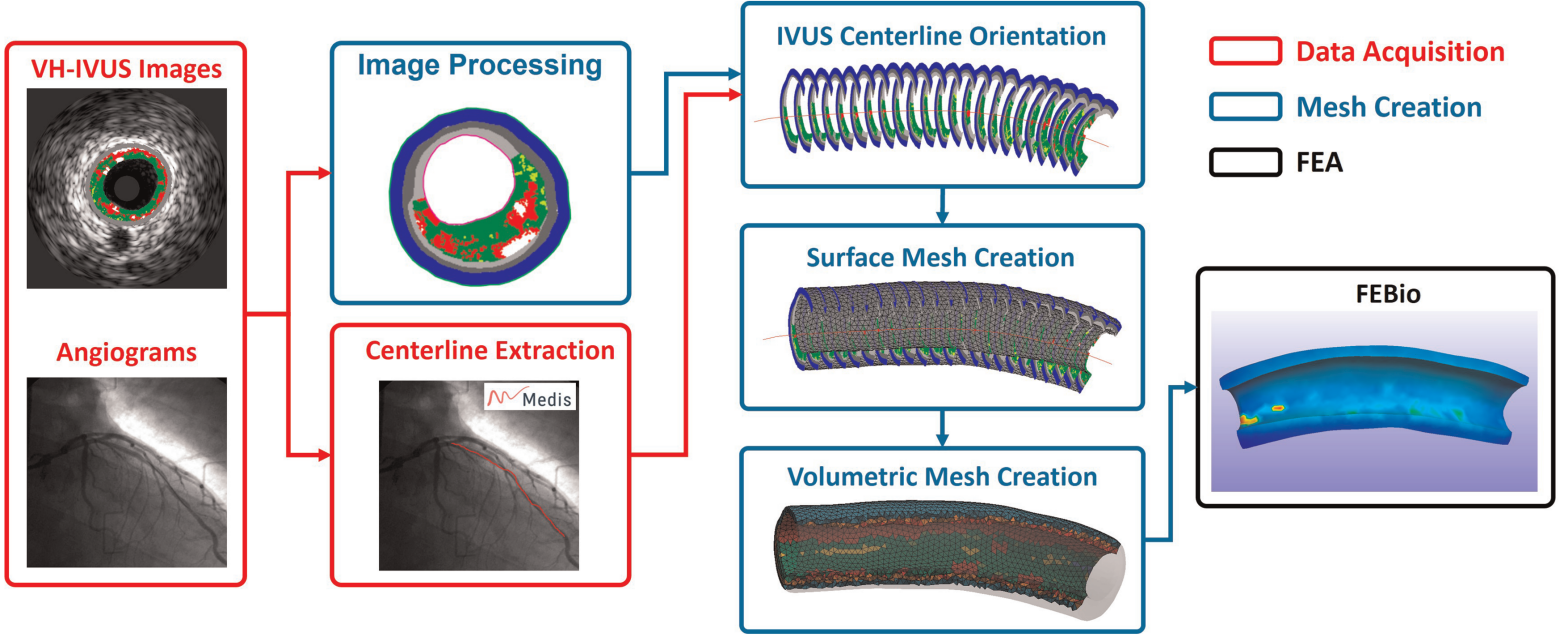
Simplified workflow highlighting the steps in creating a patient-specific artery model with material properties. Artery centerline is extracted from angiogram data (Red). VH-IVUS images are processed to extract individual plaque constituent data, oriented along the 3D centerline, and meshed to create the volumetric finite elements (Blue). FE analysis is solved in FEBio (Black).

The methods presented in the following subsections outline our approach for automatically generating volumetric meshes using VH-IVUS images and coronary angiography data (Figure 1). The algorithm was developed using the MATLAB (R2022a) scripting language and a 11-artery dataset, from 11 patients, consisting of coronary VH-IVUS images and angiography data. The script and user interface allows the user to modify meshing parameters of the arterial geometry as well as simulation parameters for FEBio.

### Data acquisition

**Figure 2:**
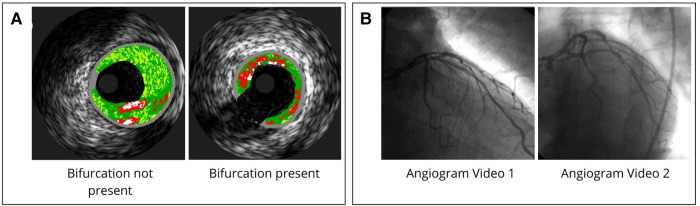
VH-IVUS and angiogram data. (**A**) Example of VH-IVUS images comparing bifurcated and non-bifurcated areas. (**B**) Angiogram data acquired from coronary angiography videos from two perspectives.

A dataset of twenty-seven patients between 2007 and 2009 with moderate coronary artery disease (CAD) enrolled in an Atorvastatin clinical trial at Emory University (ClinicalTrials.gov; Identifier: NCT00576576) (15, 16). All patients received optimal medical therapy for cardiovascular risk factors, including 80 mg atorvastatin daily. According to standard of care, all patients underwent baseline and 6-month follow-up biplane coronary angiography as well as EKG-gated (R-wave peak) radiofrequency backscatter virtual histology-IVUS (VH-IVUS) image acquisition of the proximal left anterior descending (LAD) and left main (LM) coronary arteries (20 MHz Eagle Eye® Gold Catheter, Volcano Corp., Rancho Cordova, CA). Clinically, angiography provides 3D geometry and indicates areas of stenosis of the artery, while IVUS imaging enables visualization of the properties of the artery wall. Virtual Histology (VH, Volcano Corp.) converts the IVUS radiofrequency spectrum to construct a color-coded tissue map overlay on the IVUS images. The tissue map consists of four materials: fibrous tissue (densely packed collagen fibers), fibrofatty tissue (loosely packed collagen fibers with minimal lipid deposition), dense calcium (calcium deposits), and necrotic core (high lipid content and areas of necrosis) (Figure 2) (17–19). VH-IVUS images were acquired at an automated motorized pullback (0.5 mm/s) from approximately 60 mm down the LAD up to the guide catheter in the aorta. Lastly, Doppler derived pressure data was acquired in the LM and distal LAD coronary arteries using a 0.355 mm monitoring guidewire (ComboWire, Volcano Corp.). Of note, none of the patients in the trial experienced a plaque rupture or MACE after 6-months.

After approval by Emory University and UT Dallas Institutional Review Boards, data collected in the clinical trial was used for FEA analysis. Of this dataset, for the development of our methods, we used arteries from eleven different patients who exhibited varying degrees of tortuosity, plaque heterogeneity, and number of branches to best represent the range of inputs that may be seen in the clinic. Hence, we will refer to these arteries as numbered from one to eleven for the remainder of this manuscript. Analyzed arteries excluding arteries 8, 9, and 11 have bifurcations present. Patient specific diastolic and systolic pressure values are recorded in Supplementary Table S1. The mean systolic pressure across the dataset of 17 kPa (131 ± 24.8 mmHg) was used for each arterial model (Supplementary Table S1). Lastly, 3D centerline coordinates were extracted from the angiogram videos using QAngio (20) and exported for use in the mesh creation process (Figure 2).

### Mesh creation

#### VH-IVUS image processing and centerline orientation

**Figure 3:**
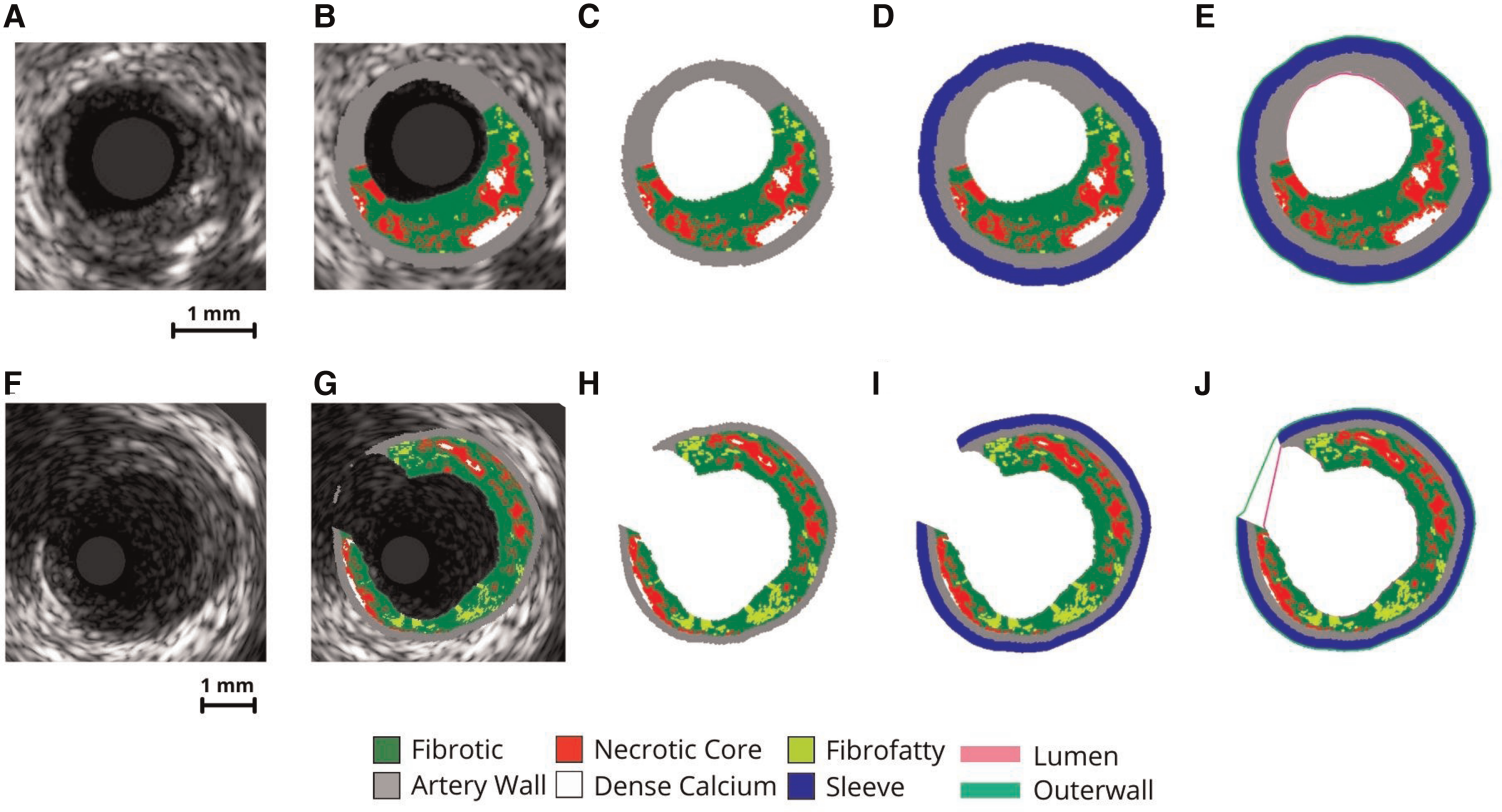
VH-IVUS imaging processing procedure. Image processing steps of VH-IVUS images with (**F-J**) and without (**A-E**) a bifurcation. (**A,F**) raw IVUS images. (**B,G**) VH overlay on the raw IVUS image. (**C,H**) image after extracting plaque constituent pixels. (**D,L**) image after adding sleeve layer. (**E,J**) Image after extracting inner (lumen) and outer surface profiles.

Generic IVUS imaging produces grayscale images (Figures 3A,F) which are then processed using virtual histology (Figures 3B,G) and exported. These VH-IVUS images are filtered to remove background noise and separated into material groups depending on pixel RGB values (calcium, necrotic core, arterial wall, fibrofatty, and fibrous) (Figures 3C,H). These filtered images are then masked, and islands are removed using an area-opening algorithm (21).

To create a support layer of tissues for protecting thin arterial walls, a perivascular “sleeve” layer with a predefined thickness (0.2 mm radially) is added (Figures 3D,I) and the lumen and outer surface profiles are extracted (Figures 3E,J). The sleeve layer acts as a buffer and was implemented for two reasons. First, it allows the creation of volumetric elements where the artery is too thin. Second, it provides an external buffer for the artery wall to push against during pressurization, ensuring model stability during the simulation. Pixel coordinates and profiles are then scaled to millimeters (1 pixel = 0.02 mm) and oriented in 3-D along the centerline. In the case of bifurcated arterial segments, an additional step is used to obtain the branched geometry. Here, the code performs a radial sweep to find discontinuous IVUS pixels and identify where a branch occurs within the inner and outer profiles. These images are flagged and the inner/outer profile coordinates corresponding to the branched gap are stored for use in the surface mesh creation step.

#### Surface mesh

**Figure 4:**
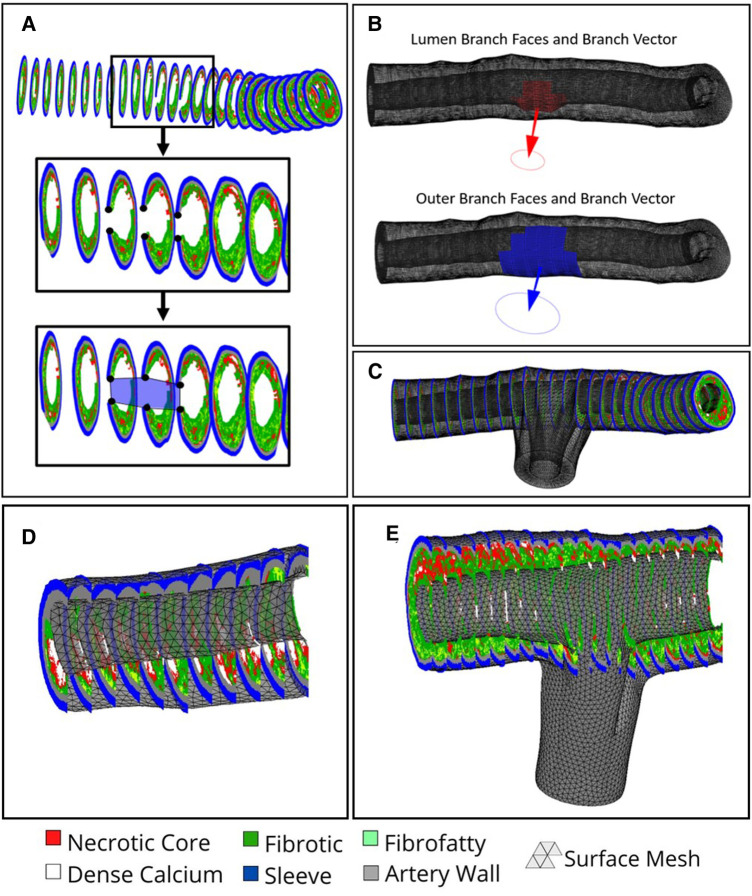
Branch creation procedure. (**A**) Black dots overlayed on VH-IVUS image represent the inner and outer coordinates proximal to the branch region in blue. (**B**) Computed Normal vectors to plan the branch lofting procedure. (**C**) Branched mesh post lofting procedure merged with main arterial mesh. (**D**) non-bifurcated and (**E**) bifurcated arterial meshes, respectively, after smoothing and resampling is applied.

The luminal and outer surface profiles extracted from the VH-IVUS images are used to create a first pass approximation of the arterial mesh *via* procedural lofting. The ends of the arterial mesh are closed by lofting from the outer edges to the luminal edges. In the case of bifurcated arteries, IVUS images containing a branch are flagged and the luminal/outer profile coordinates within the branch are stored (Figure 4A). These profiles are still used to create the first-pass approximation of the mesh and hence, the nodes correlating to the branched regions are easily identified. The faces in the mesh connected to the branch nodes are removed and the resulting holes are used to identify edge sequences for branch creation. The coordinates along these edges are averaged for the luminal and outer surfaces separately and subtracted to create a normal vector between them, defining the branch angle (Figure 4B). This branch angle is then used to project the two edge sequences (luminal and outer) onto an orthogonal plane at a predefined distance offset from the outer surface of the mesh. Here, the two edge sequences will typically overlap, causing problems when lofting to the projected edges. Hence, the edges along the luminal branch edge sequence are dilated and projected back onto the mesh wherein the faces on the outer surface that lie inside the projected space are removed. Such an assumption is blind to the true thickness of the branched artery wall; however, it is necessary since the thickness of the branched artery wall cannot be determined from the axial VH-IVUS images. Next, we loft the branch edge sequence to the projected nodes *via* a spline loft. The spline loft requires additional computation of start and end vectors parallel to the mesh faces and orthogonal to the projection plane, respectively. Once complete, the branch is closed by lofting the outer edges on the projected plane to the luminal edges on the projected plane (Figure 4C). This procedure is completed for each branch and results in a closed-volume first-pass approximation of the arterial mesh. Though this first-pass approximation resembles the real arterial structure, it comprises of non-uniform triangles and significantly varying edge lengths. Hence, we apply Humphrey class smoothing (22) and a resampling algorithm to create the uniform triangular mesh (Figures 4D,E).

#### Volumetric mesh

The final step in the mesh creation procedure is to generate the volumetric mesh *via* tetrahedralization in preparation for FEA. Currently, our method uses linear tetrahedral-4 (tet4) element shapes, although the user interface allows for nonlinear tet10 element shapes to be used instead. The decision to use tetrahedral element shapes was informed by (23) which pointed toward tetrahedral elements as the most applicable shape for biomechanical loading and use with nonlinear material models. Although other element shapes exist, tetrahedral elements allow for automated utilization of the open source tetrahedralization code TetGen (24), implemented within the GIBBON library (25), to automate the creation of the finite elements. Additionally, TetGen allows the use of command line flags to control the various optimization parameters used when tetrahedralizing the mesh. Here we use a maximum volume constraint paired with an edge length constraint to maximize element quality and produce elements with uniform volumes and edge lengths. The values of each constraint are automatically calculated in our code using the mean edge length of the surface mesh as well as a user-defined mesh resampling resolution.

### Material assignment

As shown in Table 1, each artery is composed of 5 types of materials: arterial wall, fibrotic, calcium, necrotic core, and sleeve. Materials were assigned based on their specific VH-IVUS color (i.e., RGB value). The material properties were assumed to be linear elastic as suggested by previous studies (26).

**Table 1:** Material properties used for FEA. VH-IVUS color, associated Young's modulus (MPa), and Poisson's ratio for each material type used in the FEA simulations (26).

Material	Color	Young's modulus (MPa)	Poisson's ratio
Arterial Wall	Grey	0.3	0.48
Fibrotic	Dark Green	0.6	0.48
Fibrofatty	Light Green	0.5	0.48
Calcium	White	10	0.48
Necrotic Core	Red	0.02	0.48
Sleeve	User Defined	0.4	0.48

Up until this point, VH-IVUS images have been processed and the surface mesh and volumetric meshes have been generated. However, the finite elements have not yet been associated with a particular material type. We use the arterial centerline and VH-IVUS pixel coordinates to associate each finite element with a tissue type (Figure 8A) and the associated material properties (Table 1). To do so, the centroid of each element in the volumetric mesh is first calculated. We then loop through each element centroid, find the nearest VH-IVUS pixel coordinate, and assign the material type associated with that coordinate to the element (Supplementary Figure S3). Naturally, one can see how the computational cost of comparing millions of VH-IVUS pixel coordinates with each element centroid increases exponentially with the number of elements. Therefore, to decrease computational cost, VH-IVUS pixel coordinates and element centroids are first pre-indexed into overlapping sections along the centerline. Materials are then assigned to each element by only searching the VH-IVUS pixel coordinates within the element's section rather than searching the entire mesh domain. This approach significantly reduces the number of calculations needed, thereby decreasing the time and computational resources needed to assign materials. Preliminary analysis of this process has shown that the time taken to assign materials with this optimization technique takes ∼15 min or on average between 20–40x less time; significant reductions in memory use and computational resources allow for faster material assignment with higher resolution meshes. Once materials are assigned, the volumetric mesh can be prepared for analysis in FEBio.

### FEBio setup - loads, boundary conditions, and solver parameters

Here we outline the loads, boundary conditions and assumptions within the FE model. Since the aim of our method is to model the general structural mechanics within the artery wall, the loads, BCs, and assumptions presented herein are not geared toward a specific model, but rather to provide a base framework for future modification and improvement. An overview of only the necessary conditions for general modelling of arterial mechanics is provided.

#### Luminal loading

**Figure 5:**
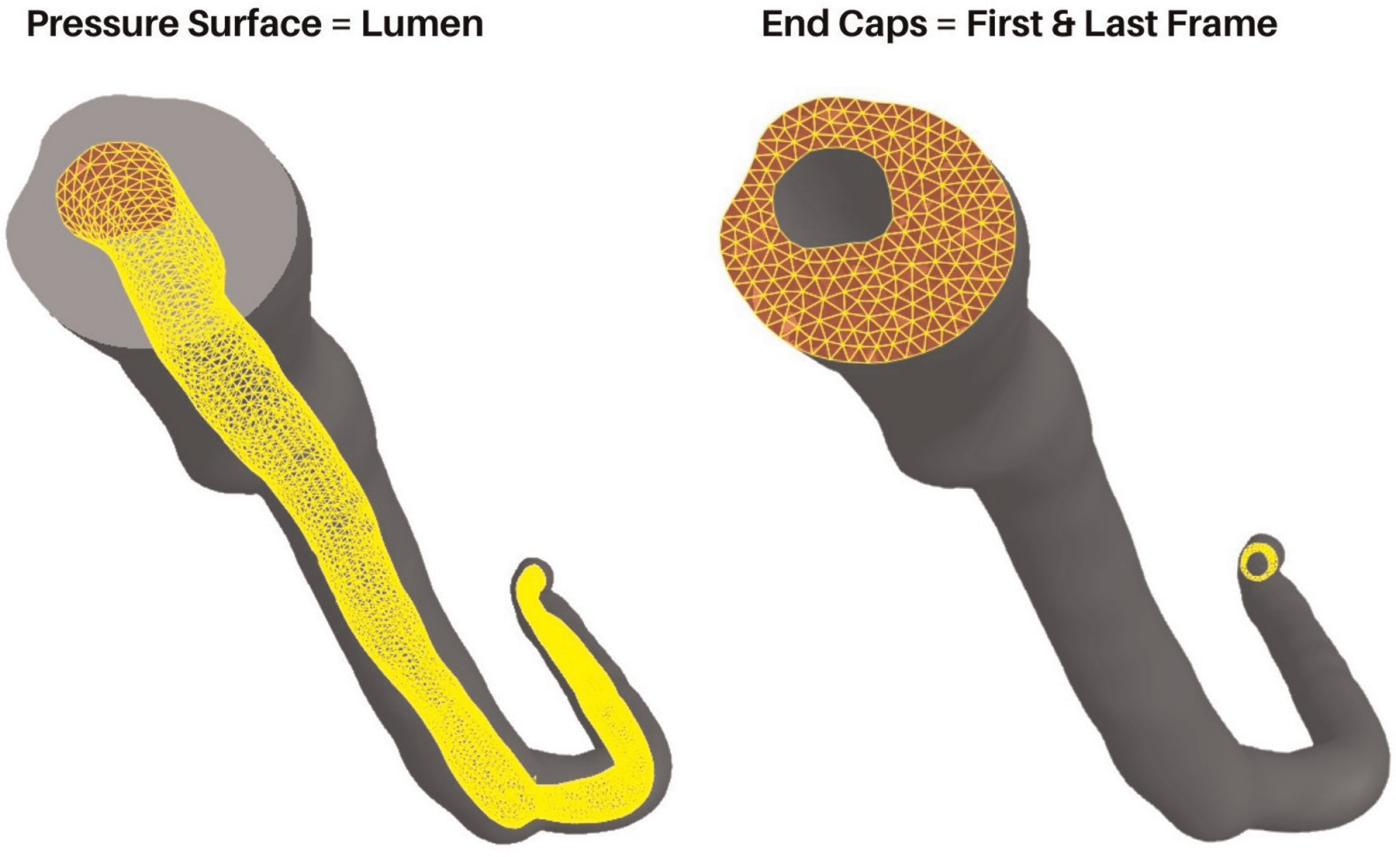
Boundary conditions and loading. The luminal surface (Left) is used to apply pressure loads and the end caps (Right) are fixed in x, y, and z axes.

Loading conditions replicate in-vivo conditions with physiologically relevant pressure loads defined at the faces of the lumen surface (Figure 5, left). In the current version of the model, a static pressure load representing systolic blood pressure is used. However, this value is user-defined, allowing the use of patient-specific pressure values or, with slight modification, the incorporation of a time-dependent load curve to be used. Pressure loads are defined at the mesh faces but FEBio distributes these values to the associated nodes internally. The pressure is applied over the course of one second with a small time-step to capture the small deformations and ensure model convergence. Here it must be noted that although the goal of our method is a simplified general model, care must be taken when choosing the time step and pressure loads. Larger pressure loads will inherently result in larger deformations and hence, a smaller time step must be chosen. Additionally, it is worth noting that a static load does not represent the dynamic pulsatile loading exhibited in-vivo. However, as previously mentioned, we aimed to create a basic framework to model general arterial biomechanics without introducing any unnecessary complexity in the current iteration.

#### Boundary conditions and assumptions

As is the case with all finite element models, boundary conditions (BCs) must be carefully chosen by considering relevant observations and assumptions. In the case of coronary artery segments, they are not simply floating in free space nor isolated from surrounding tissues. They are surrounded by various perivascular tissues, in direct contact with the myocardium, and in-line with arterial tissue outside the scope of the VH-IVUS imaging domain. However, one can still gain useful insight into the general arterial biomechanics by using simplified boundary and loading conditions. Therefore, BCs were chosen to ensure model convergence while replicating pseudo-physiological conditions. Nodes connected to the surfaces at the ends of the main artery line as well as the ends of the branches are fixed in all axes (Figure 5, right). Rotational, compressive, or tensile forces generated by heart contraction were not considered. Another factor not considered are residual stresses present in the artery in-vivo. The advent of bi-axial artery tissue testing and the opening-angle theory present in arterial continuum mechanics models made it clear that residual stresses are always present in the vasculature. For uniquely heterogenous tissues, these factors, although widely studied and published since the 1990s (27–29), require additional experiments and procedures that are difficult to conduct without destroying the tissue itself. Therefore, we do not consider residual stresses in our model and focused solely on the biomechanics at play during pressurization.

## Results

Our algorithm is capable of automatically generating a biomechanical model from patient-specific VH-IVUS images and angiograms. The algorithm was developed using MATLAB scripting language and open-source programs such as TetGen and FEBio. Depending on the number of IVUS slices, meshing resolution, curvature complexity, type of computer, etc., the time taken to reconstruct a mesh will vary broadly. For example, a mesh reconstruction with 200 frames and 0.2 resolution took around 15 min on a generic consumer laptop. User-friendly step-by-step instructions as well as the source code are available on GitHub: https://github.com/VMBL-UTD/Automated-Artery-Reconstruction.

### Mesh convergence study

A mesh convergence study was conducted to determine the resolution that best captured the volumes of each plaque constituent. Although the speed and power of computer processors only increases with time according to Moore's law, it is still worth considering such a mesh convergence study to minimize computational resource consumption and problem complexity. The mesh resampling value is a user-defined parameter and refers to the triangular and tetrahedral edge lengths (in millimeters) of the surface and volumetric meshes, respectively. Hence, within this section the terms “resampling value”, “edge length”, and “resolution” are used interchangeably.

**Figure 6:**
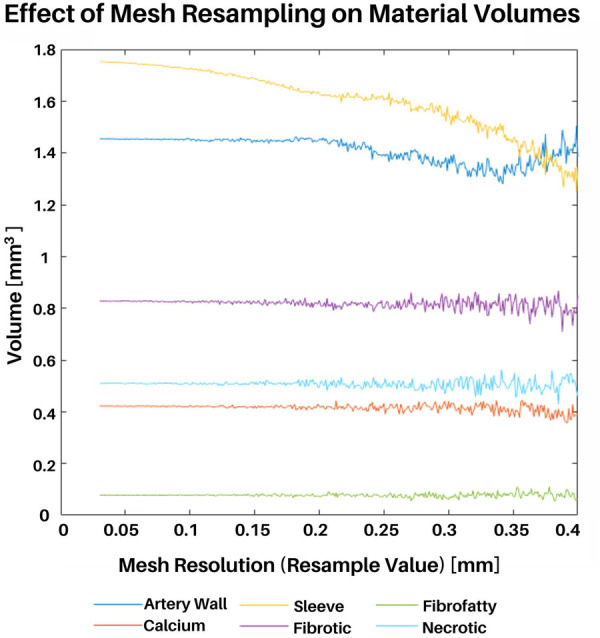
Mesh convergence study. An entire representative artery was reconstructed at various mesh resolutions. Meshing resolutions ranging from 0.04 mm to 0.4 mm in steps of 0.005 were used (x-axis). The total volume of each plaque constituent was calculated at each resolution (y-axis).

For this study, a representative full artery mesh was reconstructed using resampling values ranging from 0.04 mm to 0.4 mm in steps of 0.005 mm. Additionally, the total volumes of each plaque constituent (medial, adventitial, necrotic, calcium, fibrotic, and fibrofatty) were calculated at each resampling value (Figure 6). It is also worth noting that although the sleeve element volumes were also considered in this study, mesh convergence was primarily determined by the actual plaque constituents taken from VH-IVUS data. Coarser mesh resolutions (larger resample values) resulted in inconsistent variations in constituent volumes, whereas finer meshes (smaller resample values) saw the convergence of constituent volumes. Edge lengths above 0.2 mm resulted in varying constituent volumes with increasingly inconsistent volumes; going from a resampling value of 0.3 to 0.35, for example, resulted in large differences in fibrotic element volumes. Edge lengths between 0.2 mm and 0.04 mm produced constituent volumes that were largely unchanged, hence, for the purpose of the following subsections and to save computational resources, a value of 0.2 mm was used. The mesh convergence results verify that the model behaved as expected (i.e., finer resolutions between 0.04 mm to 0.2 mm, resulted in more precise constituent volume).

**Figure 7:**
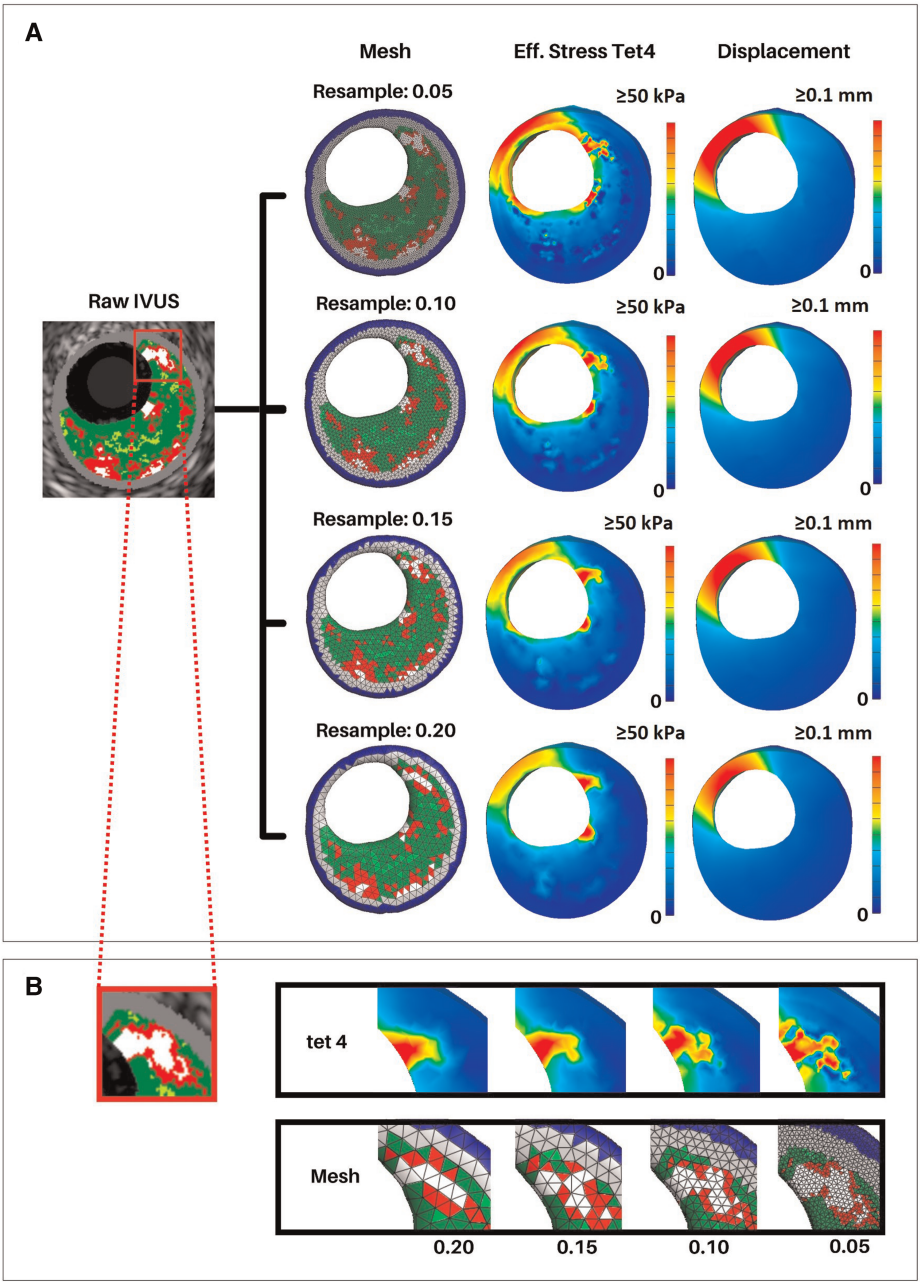
Effective stress and displacement with varying mesh resolutions. Arterial meshes of a short 5 VH-IVUS slice artery segment were reconstructed at resolutions of 0.05, 0.1, 0.15, and 0.2 mm. Similar simulation parameters were used in all meshes to calculate effective stress and displacement in FEBio. (**A**) First column shows the middle slice of the reconstructed meshes. Second and third columns show cross-sectional views of the effective stress and displacement, respectively. (**B**) Close-up view of a calcified region of the middle VH-IVUS image and corresponding regions of the arterial mesh. The top row shows effective stress, and the bottom row shows the mesh at each resolution.

Once the meshing resolutions that best captured plaque constituent volumes were identified, we conducted a stress-based convergence study to quantify the impact of mesh resolution on stress distribution (Figure 7, Supplementary Table S2). This study was designed to show how the distribution of effective stress, displacement, and element size change with different mesh resolutions as compared to the original VH-IVUS images. A representative section of the artery was reconstructed using 5 IVUS images at resolutions of 0.2, 0.15, 0.10, and 0.05 mm and the same simulation parameters, as outlined in “FEBio setup - loads, boundary conditions, and solver parameters”, were utilized. Owing to the fixed boundary conditions (in x, y, and z) applied to the nodes at the end caps, peak stresses were in the elements nearest the end caps. Hence, we chose to use a cross sectional view from the middle of the segment where the fixed boundary conditions would have less of an effect on the stress distribution. Regarding deformation, the thinnest region of the cross-section consistently had the largest displacement magnitude as expected. Maximum displacement values gradually increase with finer resolution (i.e., maximum displacement increases from 0.11 to 0.14 mm at resolutions 0.2 to 0.05 mm, respectively). Volumetrically, the size of the elements decreased linearly with smaller edge-length values which aligned with the outcomes of the volumetric mesh independence study. Mechanically, effective stress in the artery also decreased as the resolution neared a 1:1 ratio with the VH-IVUS pixel size. This pattern can be attributed to a shear locking phenomenon typically experienced by linear element shapes wherein they are unable to accurately capture deformation in nonlinear materials. The trend of stress increasing with finer resolutions could also be attributed to our material assignment method. Though the material assignment method does apply similar materials globally, larger meshes resulted in less consistent local material assignment. This discrepancy arises because our current approach uses element centroids to find the nearest VH-IVUS pixel for material assignment. Due to the image pixel edge-length value being significantly smaller than the mesh, a neighboring pixel could be nearer to an element's centroid when a slightly different resolution is applied. An example of this limitation is seen in Figure 7B where the volume of the red necrotic core pixels is over approximated in larger meshes and more precisely represented with finer resolutions. Moreover, the stress distribution in Figure 7B are clearly impacted by coarser resolutions. This result paired with the plaque heterogeneity seen in the VH-IVUS image suggests that finer resolutions are better suited for a more precise representation of the VH-IVUS pixels. Furthermore, we found that using nonlinear tet10 element shapes results in more consistent stress calculations compared to linear tet4 elements at the same meshing resolutions (Supplementary Figure S4). Supplementary Table S3 quantifies the percent difference in stress values as a function of element shape and mesh resolution for each material type. The user interface (GitHub repository: https://github.com/VMBL-UTD/Automated-Artery-Reconstruction) allows the user to modify the resolution and element type. Yet, one must also be aware that finer meshes can significantly increase computational time and required resources when reconstructing the mesh and running the FEA simulation.

### Volumetric differences between straight and curved geometry

We sought to determine if the volume of the plaque constituents and/or wall mechanics differed between straight and curved arterial geometry. Original works utilized straight meshes reconstructed from IVUS (32). Yet, angiogram data allows the realization of a curved centerline and hence a more anatomically accurate arterial reconstruction. One of the inputs to the mesh creation script allows the user to define whether the geometry uses the curved centerline or not. Plaque constituent volumes can then be computed from the volumetric mesh and wall mechanics from the FE simulation.

In a representative mesh, the relative volumes of the plaque constituents were 52.48% sleeve material, 17.78% fibrotic/fibrous cap, 18.34% arterial wall, 6.47% fibrofatty, 3.21% necrotic core, and 1.71% dense calcium in the straight reconstruction. The curved centerline artery reconstruction was composed of 52.02% buffer material, 17.95% fibrotic/fibrous cap, 18.49% arterial wall, 6.57% fibrofatty, 3.24% necrotic core, and 1.73% dense calcium. Statistical analysis between the plaque constituent volumes was conducted in R (version 4.1.3). Simple correlation test (*p* < 0.05, R = 0.99) shows there is a high correlation between the constituent volumes. Thus, using curved geometry instead of straight geometry does not create statistically significant differences in plaque constituent volumes.

### Effective stress

**Figure 8:**
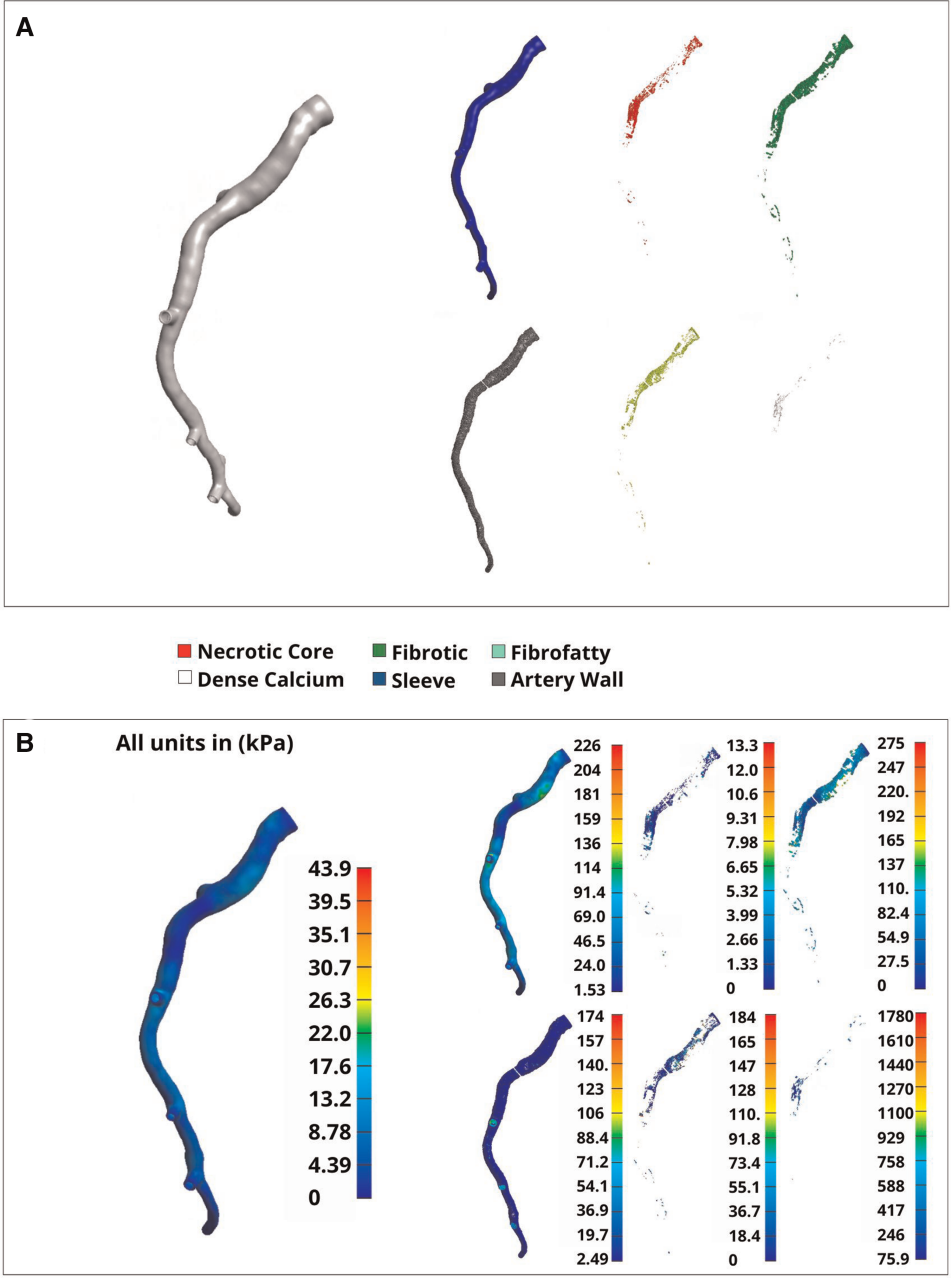
Volumetric elements and Von mises stress by material type. A full representative artery was reconstructed at a 0.2 mm resolution with tet4 elements. (**A**) Separated materials that were assigned using the plaque constituent pixel coordinates extracted from VH-IVUS images in “VH-IVUS image processing and centerline orientation”. (**B**) Nodal-smoothened effective stress across the entire artery (left) and elemental Von-Mises stress (right) corresponding to the material types noted in (**A**).

A representative arterial mesh (Artery 4) was chosen to illustrate the FEA results. Note that these results are not intended to suggest any sort of global interpretation but rather display our method's FEA capabilities. Materials were assigned according to “Material assignment” and a sleeve thickness of 0.2 mm was used (Figure 8A). Boundary conditions, assumptions, and loading parameters outlined in “Luminal loading” and “Boundary conditions and assumptions” were also used for this analysis and Von Mises elemental stresses were calculated for each plaque constituent (Figure 8B). Elemental stress was highest in calcified elements with an average of 288.17 kPa and lowest in necrotic elements with an average of 2.2 kPa. After applying nodal smoothening, the average effective stresses across the entire artery segment remained below 60 kPa. Of note, the thickness of the sleeve layer can impact simulation results and stress values. In the same representative artery, a discrete range of sleeve thicknesses from 0 (no sleeve) to 0.4 mm corresponded to a change in mean stress from 28 kPa to 19 kPa, respectively. Figures and tables displaying various mesh characteristics and results for all 11 arteries used for development of our method are included in the supplementary material (Supplementary Figures S1, S2, and Supplementary Table S1).

## Discussion

Understanding the state of stress inside atherosclerotic coronary arteries is paramount for improved patient care and treatment planning. The automated method presented herein uses patient-specific VH-IVUS images and angiogram data to produce volumetric arterial meshes. FEA is then applied to the volumetric mesh to calculate the stresses and strains that develop in the artery. A simple way to determine the biomechanics of a patient's artery will give insight into the state of a patient's disease. Previous studies have utilized VH-IVUS or OCT imaging to generate 2D planar FEA simulations of atherosclerotic plaque (26, 31, 32). Moreover, 3D FEA models of coronary arteries have also been developed, utilizing a single generalized homogeneous material for the entire artery or a limited set of heterogenous materials (33, 34). These methods provide useful insight into both planar and 3D stresses that develop within a plaque buildup during the cardiac cycle. However, the full 3D complexity of the plaque structure is impossible to be accurately depicted in planar simulations and the use of homogenous or limited materials in previous 3D simulations fail to address the complex heterogenous nature of plaque. Another factor to note is the amount of manual intervention required to generate the FE meshes in each approach. Images had to be manually cleaned and processed to extract the inner and outer arterial profiles and plaque constituents. Our method addresses each of these shortcomings, opting to combine the use of 2D images for reconstructing the mesh, 3D spatial locations of materials extracted from VH-IVUS to address material heterogeneity, and automation to limit any manual intervention. Additionally, the complex curvature of coronary arteries, captured *via* coronary angiography, is also incorporated; resulting in a method capable of automatically generating anatomically accurate arterial meshes in 3D at the press of a button.

As with all modern computational methods, the results from FEA are not definitive and results must be interpreted with care. The complex, heterogeneous nature of arterial tissue and nonlinearity of soft tissues allow only an approximate solution to be realized (35). Additionally, special care must be taken when choosing boundary conditions and loads capable of replicating physiological conditions while also minimizing computational resources and complexity. For example, fixing nodes along the end caps can result in higher stresses in adjacent elements, hence skewing the distribution of stress in nearby regions. Boundary conditions and assumptions are absolutely necessary in any computational model; however, one must also be weary of oversimplifications and misinterpreting the results. Another area requiring close attention are material properties. We currently use the widely accepted linear neo-Hookean material model presented by Paritala et. al (26), while works from Hoffman et. al (36) and Holzapfel et al. (37), have proposed Mooney-Rivlin or modified versions for atherosclerotic constituents. The nonlinear Mooney-Rivlin material model is a third-order model, which is postulated to be better suited for describing shear deformation in elastic materials (38) as compared to the second-order Neo-Hookean model currently used. However, we did not aim to quantify the difference or implications of either model but rather, offer a robust method capable of easily interchanging between material models if desired.

The stresses presented herein are within ranges seen in the literature. Work by Paritala et al. found stresses in a stenosed artery to range between 0.002 to 286.1 kPa, with a large percentage of the higher stresses focused on the plaque shoulder (26). Wang et al. reported wall stresses between 0.3634 and 450.4 kPa in planar 2D FEA simulations (39). Some variation is expected, owing to differences in materials properties or mesh reconstruction methods employed, but the stress results of our FEA lie within both ranges. Additionally, none of the atherosclerotic plaques presented herein ruptured at follow-up (16). This stability is also consistent with our results as the nodal-smoothened values from our FEA were below the suggested 300 kPa rupture threshold (40). In summary, our values are within previously published literature values.

The method presented herein aims to display the current capabilities for automated arterial mesh reconstruction, yet, as with all computational methods, this approach does come with its limitations. One shortcoming is the material assignment method. Currently, our material assignment method (“Material assignment”) simply assigns one of five materials to each element using the nearest spatial location of the VH-IVUS pixels extracted in “VH-IVUS image processing and centerline orientation”. This generic approach does not refine the transition in material stiffness at the boundary of stiff and soft materials, which may result in larger stresses in these areas (e.g., adjacent calcium and necrotic elements) as seen in Figure 7 and Supplementary Table S2. This limitation is an important and common issue in heterogeneous FE models and gives rise to the need to address material heterogeneity while minimizing additional computational resources. Of note, if desired, the user can modify the mesh resample value to address this limitation. However, to save computational resources one may consider alternative meshing approaches such as finer resolution around material boundaries or adaptive remeshing to potentially minimize numerical errors and ensure a refined transition between stiff and soft materials. Additionally, the user interface allows for manipulation of the element shape used during analysis. The code defaults to linear tet4 element shapes but nonlinear tet10 element shapes can be applied for the analysis instead; this choice will increase the amount of computational power needed for simulation and influence the biomechanical analysis (Supplementary Figure S4). A second limitation is seen in the default material model. Currently, the code defaults to using a Neo-Hookean material model which is less accurate for predicting the nonlinear response exhibited by biological tissues. This material model was chosen as a mere surrogate for the development of the algorithm but can easily be modified to use a different material model if desired. This versatility gives users the ability to define custom material models or choose from another one of the multitude of models currently implemented in the FEBio software. Although not explicitly implemented in the user interface, a custom plug-in can also be implemented with FEBio allowing for fully customized material models to be used which may be better suited for other applications. A third limitation of our method is its inability to predict plaque rupture or fully characterize plaque stability. This limitation has been a topic of interest in cardiovascular research for many years (41, 42) and a variety of predictive measures have been suggested namely, necrotic core thickness (3), fibrous cap thickness (43), and microcalcifications (5). Due to the difficulties involved with obtaining such geometrical features from VH-IVUS imaging, these aspects were not analyzed in the manuscript. However, the incorporation of different imaging modalities that are capable of identifying such structures would expand the capability of our method and enhance outcomes. In fact, the objective of this manuscript is to detail our novel, automated meshing algorithm and its potential for biomechanical analysis. We hope widespread adaptation of our method will result in valuable and insightful innovation regarding the limitations mentioned above and advancement in the realm of vascular biomechanical analysis and patient-specific precision healthcare.

In addition to acute analysis, continued development of our methods will yield novel insights into the state of cardiovascular disease. It is beyond the scope of this manuscript, but future studies can use this model to analyze the biomechanics between baseline and follow-up data to allow for more insight into the state of growth and remodeling occurring in the artery. Co-registration efforts by Timmins et al. (44) have already been used to identify how hemodynamics effects atherosclerotic progression. Such works would also allow for more insight into structural mechanical analysis as it relates to rupture mechanisms and prediction capabilities, opening the door for translational medicine and enhanced treatment options. The groundwork presented herein details an automated, user-friendly, 3D, and robust biomechanical modeling approach that has the potential to broaden research insights and utility for clinical applications.

## Data Availability

The datasets presented in this study can be found in online repositories. The names of the repository/repositories and accession number(s) can be found below: https://github.com/VMBL-UTD/Automated-Artery-Reconstruction.
