# Supplementary material for: Automated finite element approach to generate anatomical patient-specific biomechanical models of atherosclerotic arteries from virtual histology-intravascular ultrasound
Source: Front Med Technol. 2022 Nov 29;4:1008540. doi: 10.3389/fmedt.2022.1008540 (PMC9745200; doi:10.3389/fmedt.2022.1008540)
Supplement: Supplementary file 1 [file Datasheet1.docx]

Supplementary Material

# Supplementary Figures


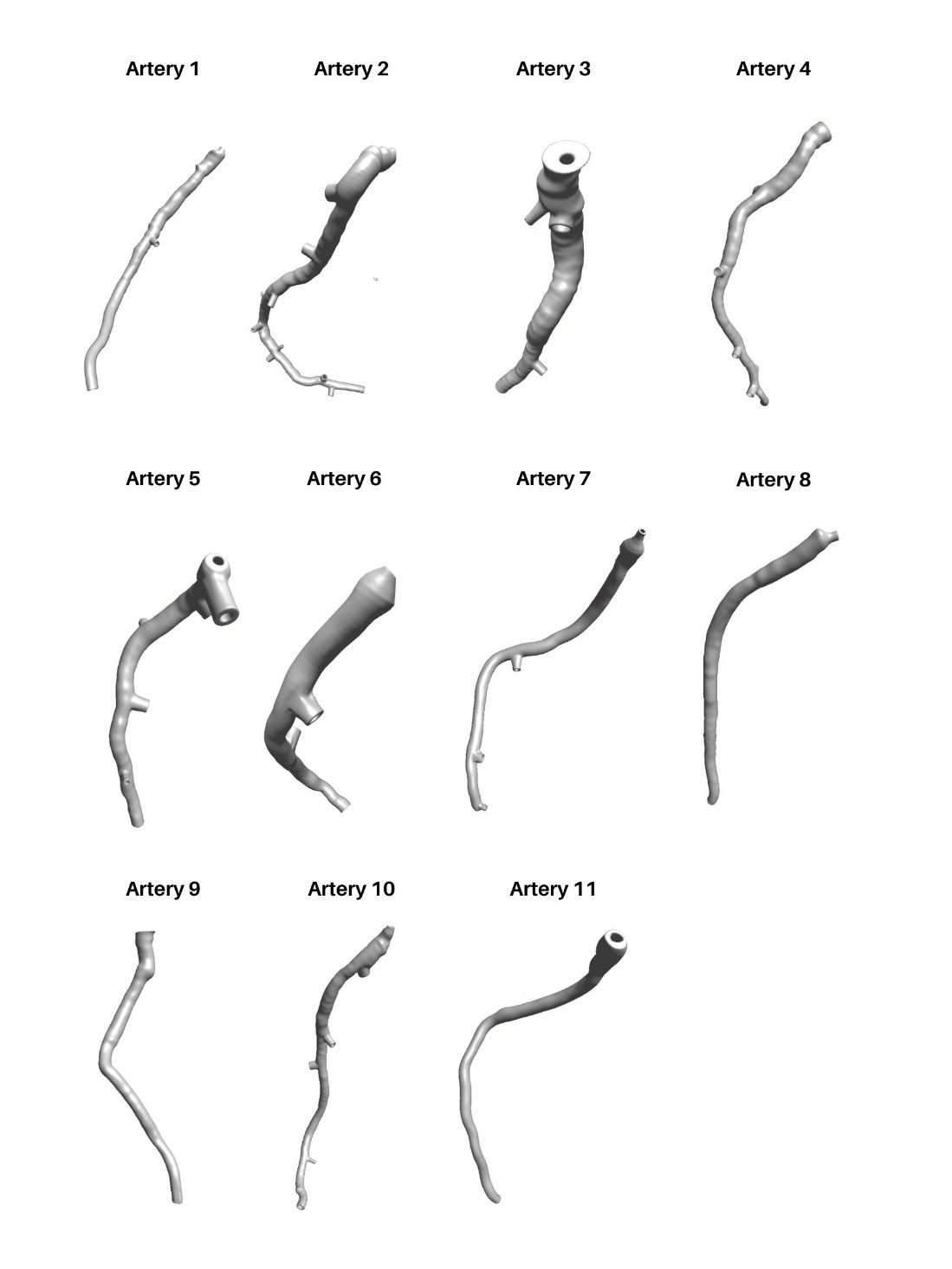
**Figure S1. Surface Mesh Reconstruction of 11 Patient-Specific Arteries.** All eleven arteries are shown with their surface meshes. Each artery was reconstructed automatically without manual interference after initialization of the input parameters.

**
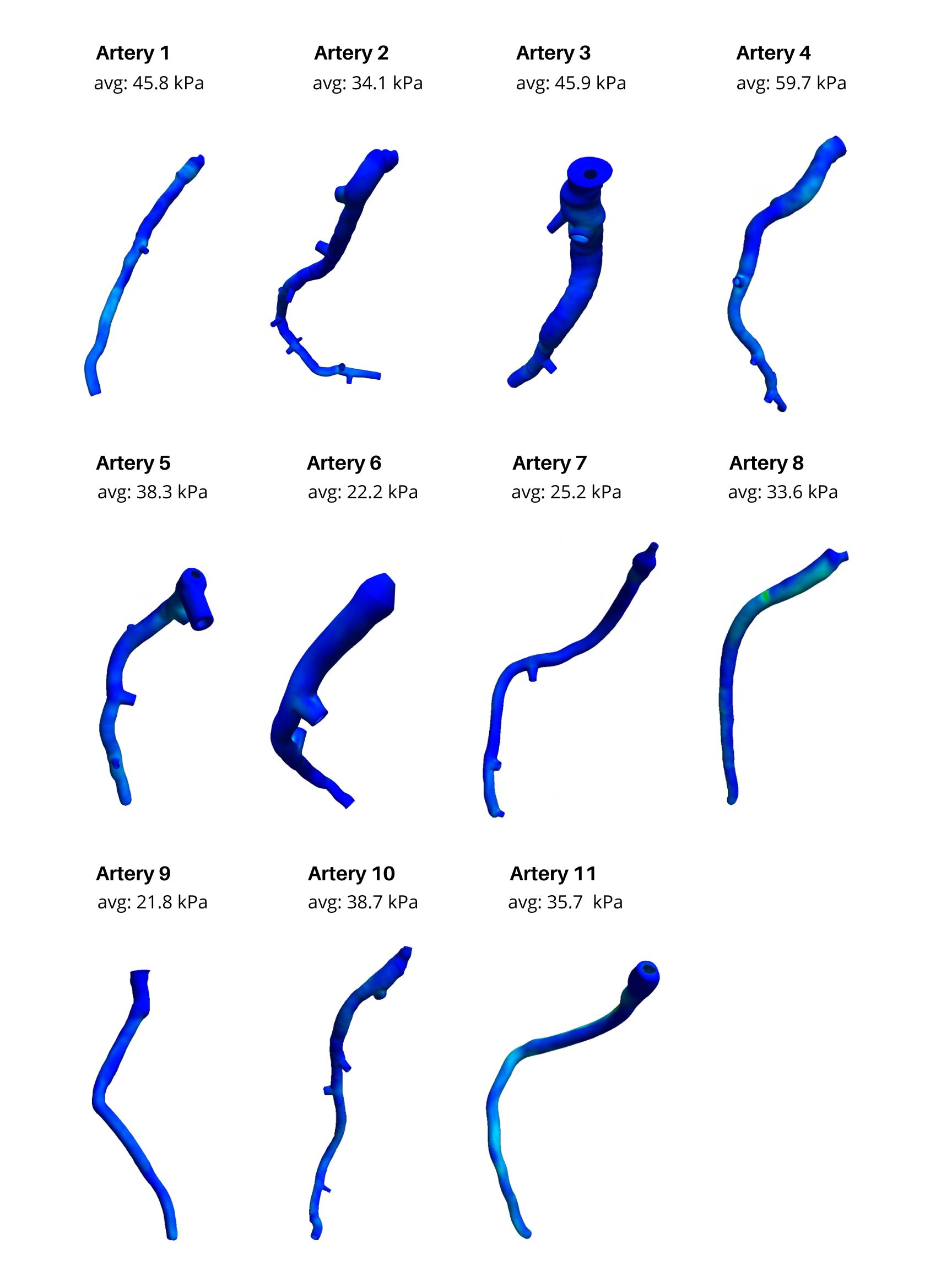
Figure S2. Preliminary Maximum and Average Von-Mises Stresses.** FEA results for the eleven arteries accompanied by the mean nodal-smoothened effective stress values.


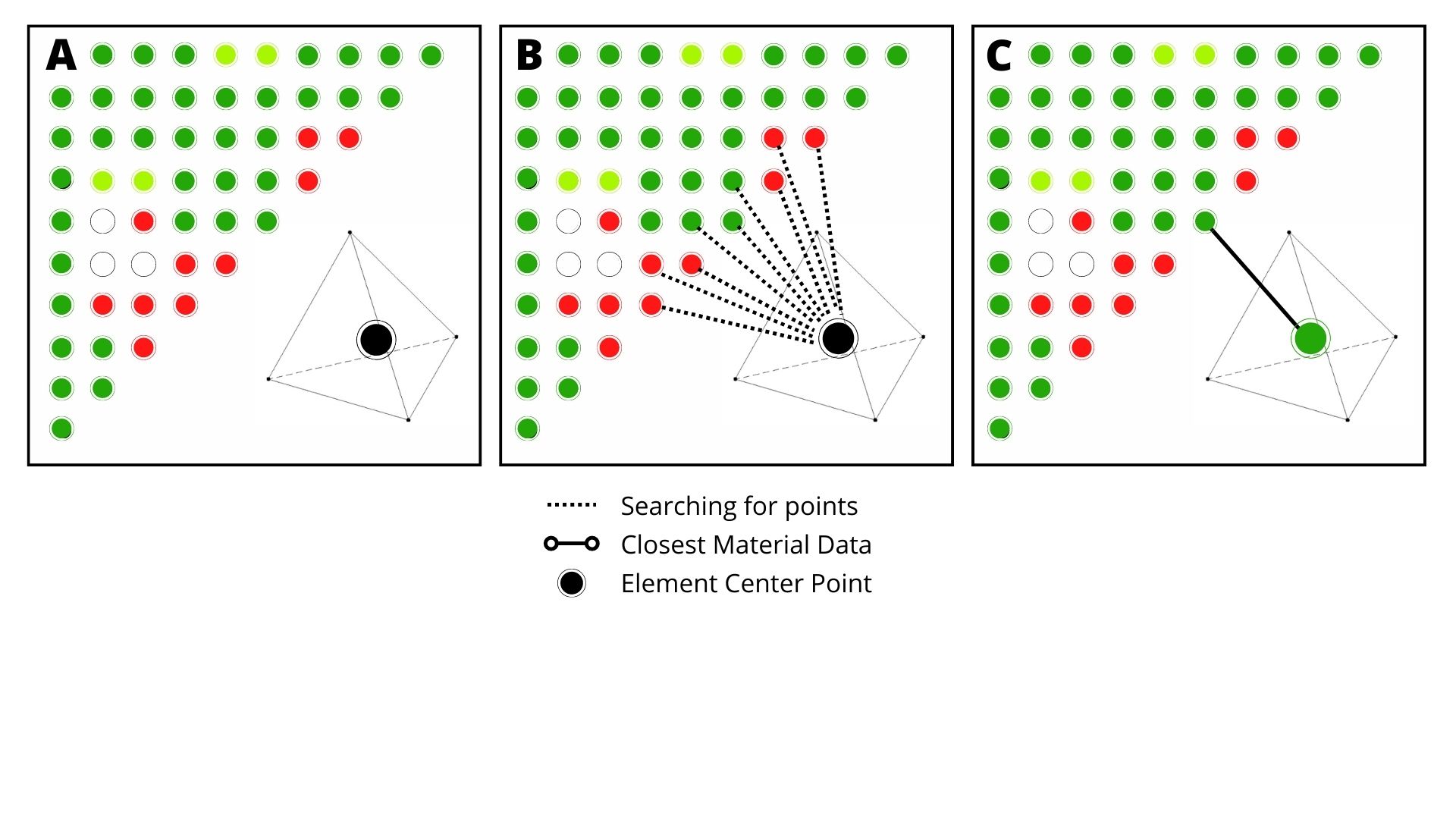


**Figure S3. Material Assignment Method.** **(A)** A single tet4 element plotted with material data along the centerline in 3D space. **(B)** The material datapoint nearest to the element centroid is found; only elements halfway between the frame before and after are searched to reduce computation time. **(C)** The element is assigned the material properties of chosen material data.


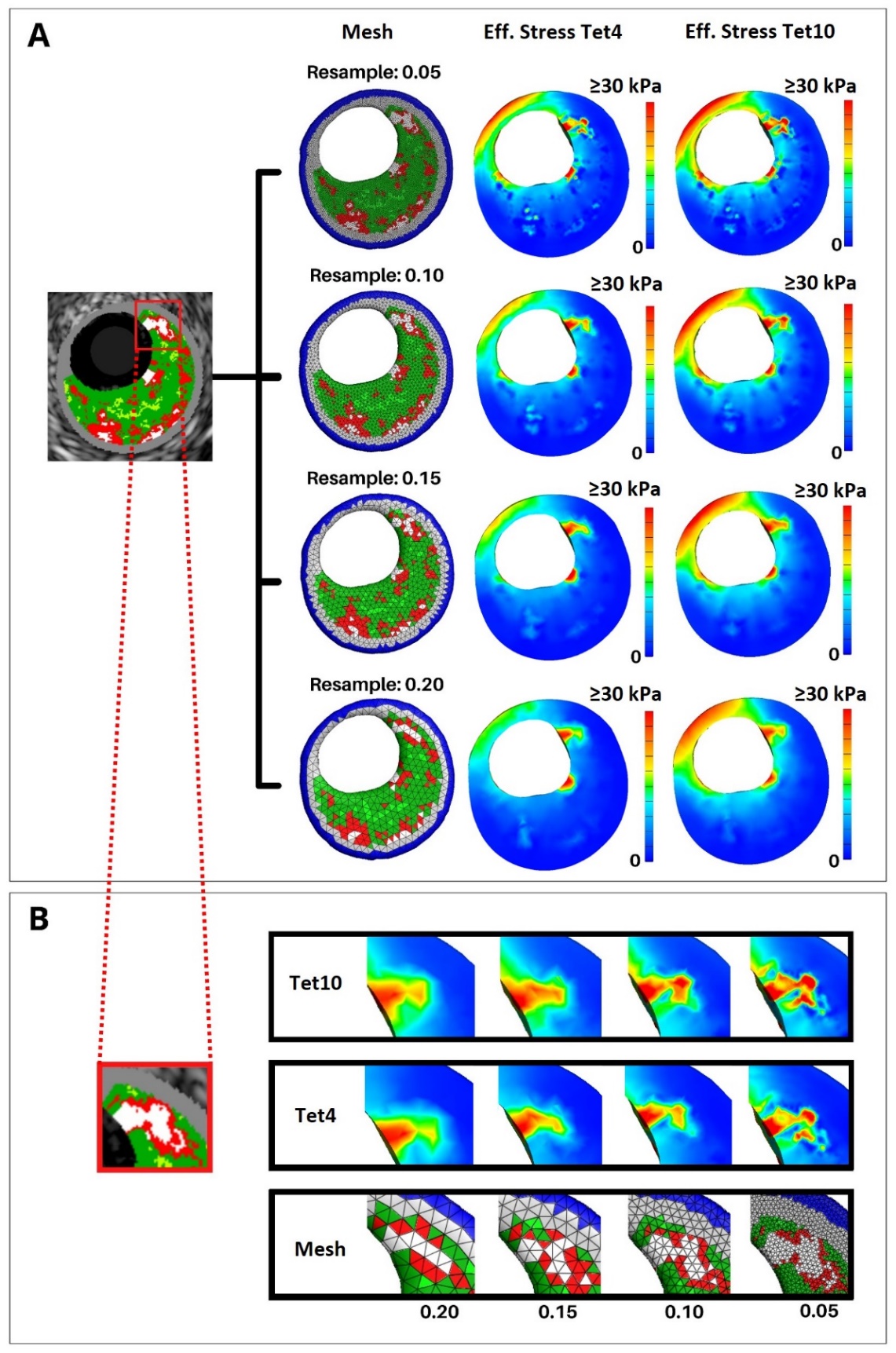


**Figure S4. Comparison Between Tet4 and Tet10 Meshes.** Meshes were reconstructed using only 3 VH-IVUS images due to issues with memory allocation when running tet10 meshes at finer resolutions. **(A)** Effective stress distributions for linear tet4 and nonlinear tet10 element shapes. **(B)** Zoomed in view of a calcified region of the mesh with effective stress for linear tet4 and nonlinear tet10 element shapes.

# Supplementary Tables

**Table S1. Mesh Properties and Preliminary Analysis Results.** Various meshing properties for all eleven reconstructed arteries. Volume composition and stresses for each material are also included.

**Table S2. Effective Stress Outputs from Figure 7.** Stress outputs at varying mesh resolutions ranging from 0.2mm to 0.05mm are shown. The maximum (Max), median, and minimum (Min) stresses were collected.

|  | **Material** | **0.2mm Resolution** | **0.15mm Resolution** | **0.10mm Resolution** | **0.05mm Resolution** |
| --- | --- | --- | --- | --- | --- |
| **Max (kPa)** | **Sleeve** | 46.14 | 51.04 | 56.29 | 61.90 |
|  | **Fibrotic** | 44.53 | 50.93 | 69.10 | 100.94 |
|  | **Fibrofatty** | 21.63 | 26.76 | 26.09 | 30.31 |
|  | **Necrotic** | 1.37 | 1.58 | 2.30 | 9.51 |
|  | **Artery Wall** | 32.71 | 37.55 | 45.13 | 55.43 |
|  | **Calcium** | 209.79 | 186.06 | 321.08 | 419.12 |
| **Median (kPa)** | **Sleeve** | 3.46 | 3.58 | 4.03 | 4.98 |
|  | **Fibrotic** | 8.54 | 10.22 | 11.06 | 12.43 |
|  | **Fibrofatty** | 7.74 | 8.95 | 9.23 | 10.30 |
|  | **Necrotic** | 0.19 | 0.25 | 0.31 | 0.46 |
|  | **Artery Wall** | 2.47 | 2.90 | 3.44 | 4.25 |
|  | **Calcium** | 52.15 | 51.26 | 47.75 | 41.83 |
| **Min (kPa)** | **Sleeve** | 0.95 | 1.10 | 1.24 | 1.88 |
|  | **Fibrotic** | 2.04 | 1.75 | 1.65 | 0.88 |
|  | **Fibrofatty** | 2.99 | 3.38 | 3.21 | 2.95 |
|  | **Necrotic** | 0.02 | 0.02 | 0.02 | 0.01 |
|  | **Artery Wall** | 0.62 | 0.58 | 0.34 | 0.18 |
|  | **Calcium** | 9.40 | 6.22 | 7.33 | 1.57 |

**Table S3. Percent Error Between Tet4 and Tet10 Elemental Stresses.** Percent error was calculated between the elemental stress in tet4 and tet10 element shapes at mesh resolutions ranging from 0.05 to 0.2mm.

| **Material** | **0.2mm Resolution** | **0.15mm Resolution** | **0.1 Resolution** | **0.05 Resolution** |
| --- | --- | --- | --- | --- |
| **Artery** | 38% | 34% | 26% | 16% |
| **Calcium** | 35% | 33% | 26% | 21% |
| **Necrotic** | 58% | 54% | 48% | 41% |
| **Fibrotic** | 38% | 31% | 24% | 16% |
| **Fibrofatty** | 42% | 35% | 27% | 19% |
| **Sleeve** | 31% | 30% | 24% | 18% |
